# Cyanobacterial Blooms Are Not a Result of Positive Selection by Freshwater Eutrophication

**DOI:** 10.1128/spectrum.03194-22

**Published:** 2022-11-29

**Authors:** Yang Yu, Wenduo Cheng, Xiaoyuan Chen, Qisen Guo, Huansheng Cao

**Affiliations:** a Division of Natural and Applied Sciences, Duke Kunshan University, Kunshan, Jiangsu, China; University of Nebraska - Lincoln

**Keywords:** cyanobacterial blooms, d*N*/d*S*, evolution, metatranscriptomics, pangenomics, purifying selection, single nucleotide polymorphism

## Abstract

Long-standing cyanobacterial harmful algal blooms (CyanoHABs) are known to result from synergistic interaction between elevated nutrients and superior ecophysiology of cyanobacteria. However, it remains to be determined whether CyanoHABs are a result of positive selection by eutrophic waters. To address this, we conducted molecular evolutionary analyses on the genomes of 9 bloom-forming cyanobacteria, combined with pangenomics and metatranscriptomics. The results showed no positive selection by water eutrophication. Instead, all homologous genes in the species are under strong purifying selection based on the ratio of divergence at nonsynonymous and synonymous sites (d*N*/d*S*) and phylogeny. The d*N*/d*S* < 0.85 (median = 0.3) for all homologous genes are similar between the genes in the pathways driving CyanoHABs and housekeeping functions. Phylogenetic support for non-positive selection comes from the mixed clustering of strains: strains of the same species from diverse geographic origins form the same clusters, while strains from the same origins form different clusters. Further support lies in the codon adaptation index (CAI) and single nucleotide polymorphism (SNP). The CAI ranged from 0.42 to 0.9 (mean = 0.75), which indicates high-level codon usage bias; the pathways for CyanoHABs and housekeeping functions showed a similar CAI. Interestingly, CAI was negatively correlated with gene expression in 3 metatranscriptomes. The numbers of SNPs were concentrated around 5 to 50. As the SNP number increases, the gene expression level decreases. These negative correlations agree with the population-level d*N*/d*S* and phylogeny in supporting purifying selection in bloom-forming cyanobacteria. In summary, superior ecophysiology appears to be acquired prior to water eutrophication.

**IMPORTANCE** CyanoHABs are global environmental hazards, and their mechanisms of action are being intensively investigated. On an ecological scale, CyanoHABs are consequences of synergistic interactions between biological functions and elevated nutrients in eutrophic waters. On an evolutionary scale, one important question is how bloom-forming cyanobacteria acquire these superior biological functions. There are several possibilities, including adaptive evolution and horizontal gene transfer. Here, we explored the possibility of positive selection. We reasoned that there are two possible periods for cyanobacteria to acquire these functions: before the onset of water eutrophication or during water eutrophication. Either way, there should be molecular signatures in protein sequences for positive selection. Interestingly, we found no positive selection by water eutrophication, but strong purifying selection instead on nearly all the genes, suggesting these superior functions aiding CyanoHABs are acquired prior to water eutrophication.

## INTRODUCTION

Cyanobacterial harmful algal blooms (CyanoHABs) are one of the most profound environmental hazards in modern history due in part to their global distribution (1), historical prevalence (2, 3), and economic consequences (4). While a variety of factors have been identified which contribute to CyanoHAB proliferation in the face of climate change (1, 5–7), many questions remain regarding their “complicated and confusing ecology” (8, 9).

To date, there remains a general consensus that nutrient loading serves as a main driver of CyanoHABs; these nutrient inputs range from macronutrients to trace metals, and nutrient residency times vary from long-term legacy nutrients to rapid ephemeral pulses (10). The corresponding metabolic pathways which utilize these nutrients in CyanoHABs have been systematically identified (11) and are curated in a web database, CyanoPATH (12). Among these pathways are physiological processes such as gas vesicle biosynthesis, nitrogen utilization, phycobilisome, CO_2_-concentrating mechanisms, and amino acid uptake. Cumulatively, these are examples of a few CyanoHAB features which are implicitly linked to nutrient acquisition and metabolism. Changes in water nutrient levels are unforeseeable to cyanobacteria; therefore, their success, observed on a global scale, attests to their superior ability to utilize elevated nutrients in eutrophic waters. From an evolutionary perspective, these superior biological functions could be acquired either prior to the onset of water eutrophication or during the eutrophication process.

In light of this recognition and the longstanding existence of CyanoHABs, one question is whether CyanoHABs are a result of positive selection by water eutrophication. In positive selection, also called Darwinian selection, genotypic changes increase the fitness of an organism (13). More specifically, either new beneficial mutations or a favorable environment enables new/existing variants to have a reproductive advantage which increases in frequency and finally fixes in the relevant population (14). In contrast, purifying selection removes (i.e., purifies) inferior variants carrying harmful mutations out of a population, typically to maintain a specific important biological function (14). Importantly, it has been shown that the widespread niches of extant cyanobacteria result from long adaptive radiation in various habitats (15). This process often involves the acquisition of some special functions in adaptation to specific habitats (16). In the presence of water eutrophication, we addressed the question of positive selection using select species of bloom-forming cyanobacteria with sufficient *in vitro* whole-genome sequences of single isolates; both molecular evolution and bioinformatics analysis were performed, including phylogeny, pangenome analysis, selection pressure, codon usage bias, and *in situ* metatranscriptome profiles in natural waters.

## RESULTS

### Discrepancy between phylogeny and biogeography in bloom-forming cyanobacteria.

**FIG 1:**
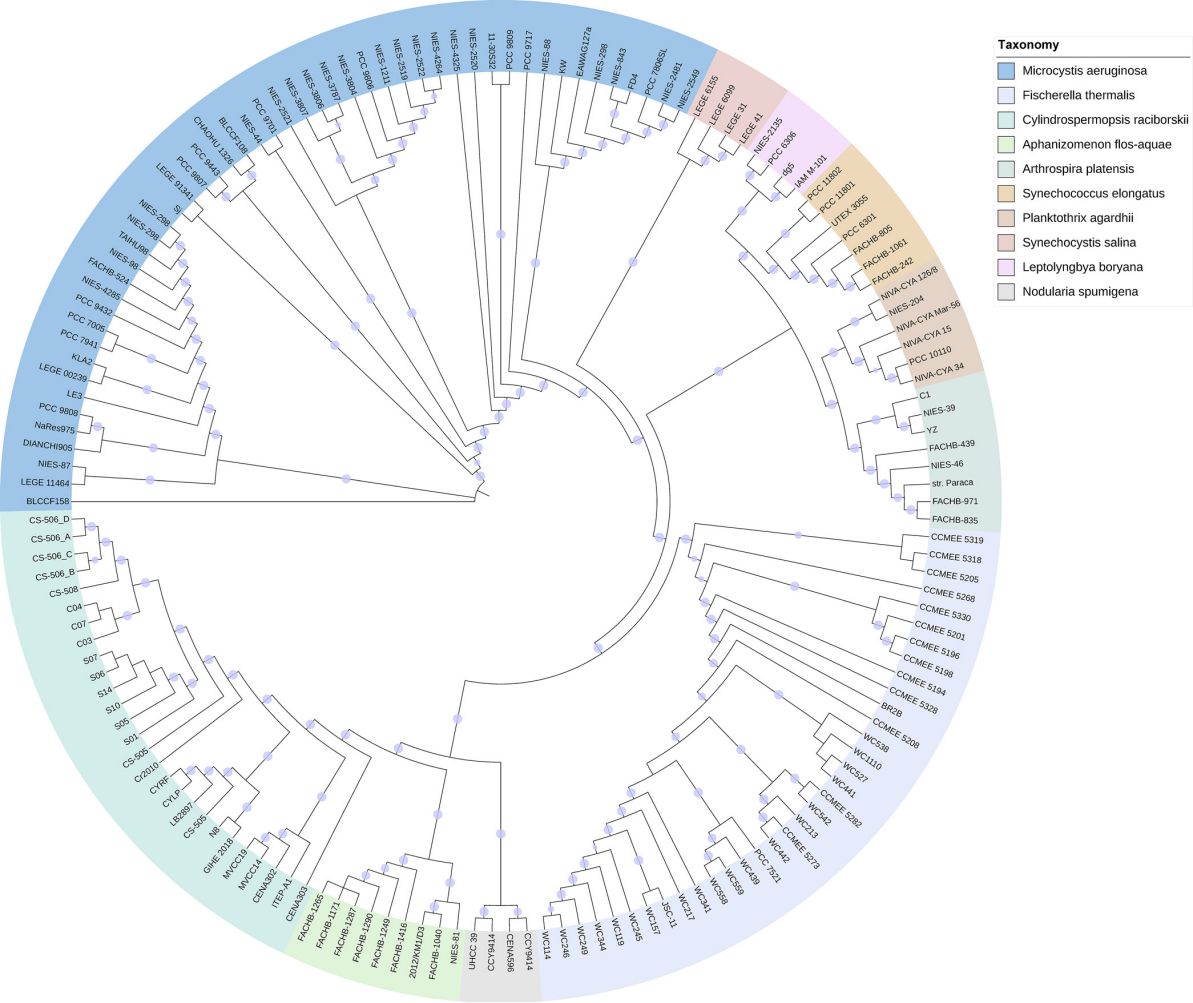
Phylogeny of 9 bloom-forming cyanobacterial harmful algal blooms (CyanoHABs) assessed in this study with *in vitro* whole genome sequences of single culture isolates. The alignment of 150 whole genomes from the National Center for Biotechnology Information (NCBI) was used to infer Maximum Likelihood phylogeny. Cyanobacteria species are color-coded, each species forming a separate clade, with *Synechocystis* spp. and *Synechococcus* spp. as reference species. Circles on the tree branches indicate bootstrap values greater or equal to 70%. Details about strains are provided in Supplemental Data Set 1.

Nine bloom-forming cyanobacterial species with at least 4 genomes of single isolates, not genomes binned from metagenomes, were included in the study. In the phylogeny of whole genome sequences, each species forms a separate clade with *Synechocystis* spp. and *Synechococcus* spp. as reference species, which have smaller genomes and earlier origins than other cyanobacteria (Fig. 1) (17). Considering the geographical origins of the strains, a clear discrepancy is observed between phylogeny and biogeography. Specifically, within the individual clades of different species, 2 types of subclades are formed: those consisting of strains from different geographic origins and those consisting of strains from the same location. Meanwhile, distances of varying lengths are clear between conspecific strains from the same locations. For example, this is most clear in the smallest clade of Leptolyngbya boryana, which comprises only four strains. One Japanese strain, NIES-2135, is closer to the European strain PCC 6306 than to the other two strains from Japan, dg5 and IAM-M101.

**FIG 2:**
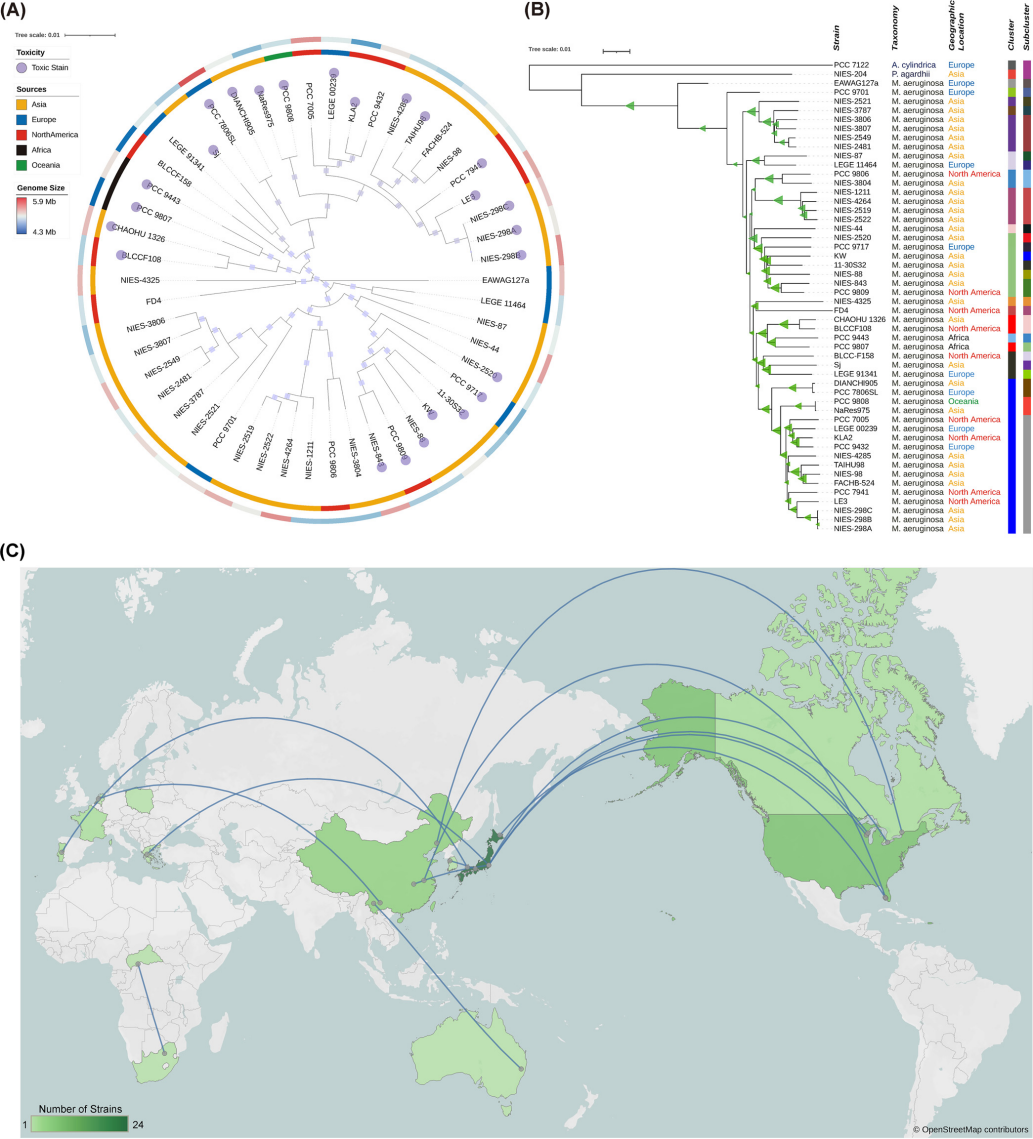
Phylogenetic trees and global distribution of Microcystis aeruginosa strains. (A) The ML phylogenetic tree built with the core genome sequences. Biogeographical location of the original culture isolate is indicated by color (Asia = orange, Europe = bue, North America = red, Africa = black, Oceanica = green) on the inner concentric circle. Genome size is shown as a color gradient from red (5.9 Mb) to blue (4.3 Mb) on the outer circle. Squares on the tree branches indicate bootstrap values greater or equal to 70%. (B) The ML phylogenetic tree built with the whole genome sequences. The tree was rooted using two cyanobacteria (Anabaena cylindrica PCC7122 and Planktothrix agardhii NIES-204) as outgroups. Taxonomy and biogeographical location of the original culture isolate of each strain are shown. Clustering was conducted using the TYGS server (22). Strains belonging to the same clusters and subclusters are marked using the same color stripes. Triangles on the tree branches indicate bootstrap values greater or equal to 70%. (C) World map showing the biogeographical location of the original culture isolate. Strains sharing the latest common ancestor are connected. Map was obtained from OpenStreetMap and is licensed under the Open Data Commons Open Database License (OdbL).

To further examine this discrepancy between phylogeny and biogeography, we focused on the largest Microcystis aeruginosa clade (Fig. 1), which has the most (18) whole genome sequences (Data Set S1). Provided with more traits associated with the strains, such as toxicity (microcystin-producing or not) and genome size, it is apparent that each cluster has strains from different geographic locations, which are often geographically far from one another. For example, one subclade has strains from Japan (NIES strains), China (TAIHU98 and FACHB strains), Europe (PCC strains), and the USA (LE3), and the other 2 subclades comprise a mix of strains from Japan, the USA, and Europe (Fig. 2B). The phylogenetically close but geographically distant strains are visualized in a global map (Fig. 2C), and at least 10 pairs of strains ranged across 5 continents. Interestingly, the toxic strains tended to cluster within a few clades, despite their origins across the 5 continents. Genome sizes tend to vary within each clade whether they consisted of international or national strains. This geography-phylogeny disparity contradicts the expectation for divergent evolution and more specifically suggests that no diverging adaptation to local eutrophic waters is required for these strains to form blooms across the world. Put differently, they are capable of forming blooms in eutrophic waters anywhere.

### Core and pangenomes analysis in bloom-forming cyanobacteria.

**FIG 3:**
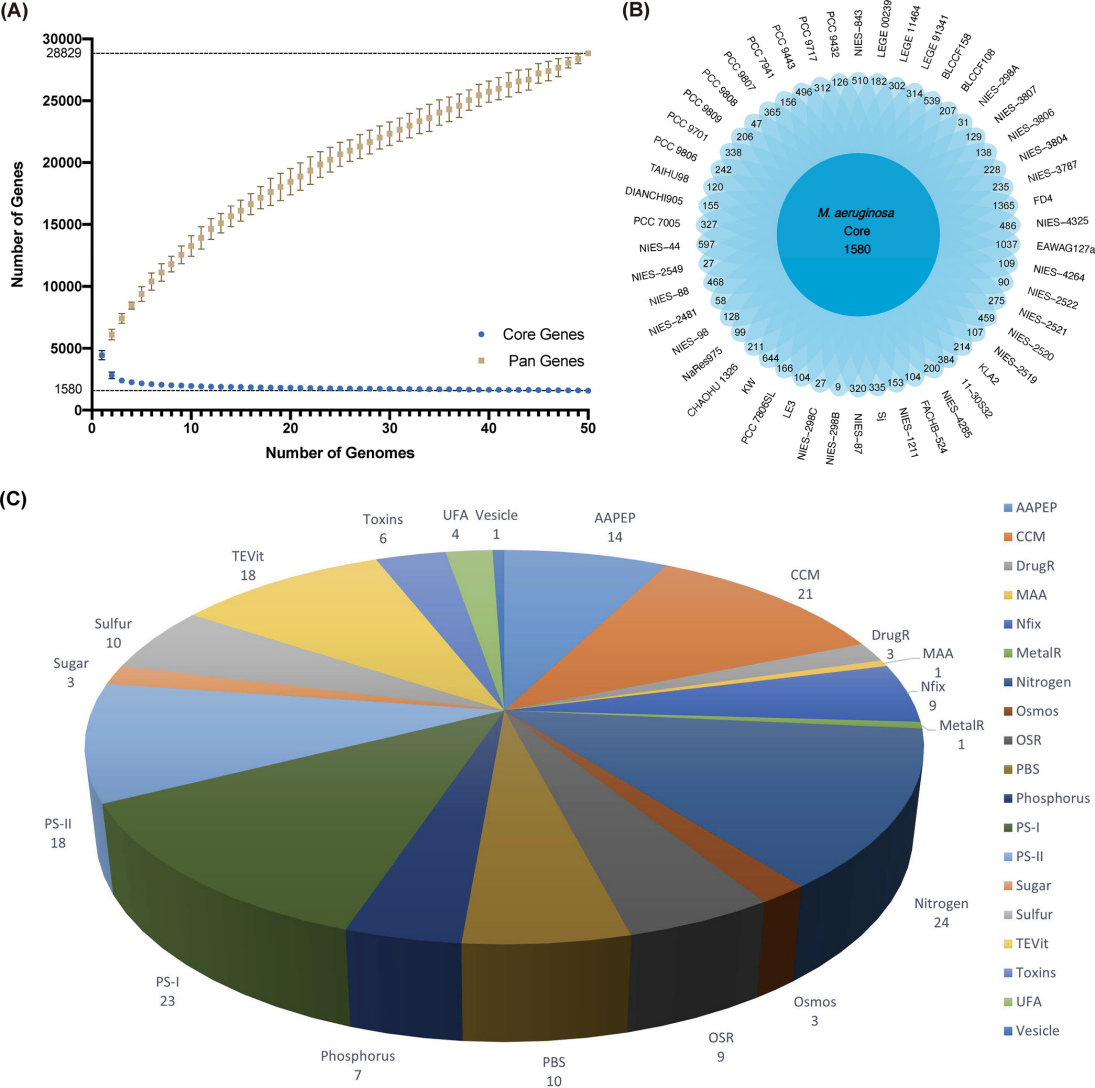
Pangenome analysis of 50 M. aeruginosa strains. (A) Core-pan-genome profiles of 50 selected *M. aeruginosa* genomes. Colored boxes denote the pan-genome (yellow) and core genome (blue) sizes, respectively. (B) Flower plot showing core and unique genes of the 50 selected *M. aeruginosa* genomes. Central circle represents the number of genes shared by all strains, while petals represent the number of unique genes in each strain. (C) Core genes of *M. aeruginosa* strains in CyanoPATH. AAPEP, uptake of amino acids and peptides; CCM, CO_2_-concentrating mechanism; DrugR, antibiotics resistance; MAA, mycosporine-like amino acid biosynthesis for screening UV radiation; MetalR, heavy metal resistance; Nfix, nitrogen fixation; Nitrogen, nitrogen utilization; Osmos, osmosis homeostasis; OSR, oxidative stress resistance; PBS, phycobilisome; Phosphorus, inorganic/organic utilization; PS-I/PS-II, photosystem I and II; Sugar, sugar assimilation; Sulfur, sulfur utilization; TEVit, assimilation of trace metals and vitamins; Toxins, cyanotoxins; UFA, unsaturated fatty acids; Vesicle, gas vesicles.

Pangenome analysis of all the bloom-forming species showed 2 general patterns: the number of total genes increased while the number of core genes decreased as more genomes were included (Fig. 3A and Fig. S1). For example, 50 Microcystis aeruginosa genomes give a total of 28,829 genes, and among them, there are 1,580 core genes (shared by all 50 strains) and 13,881 unique genes (present in only 1 strain) (Fig. 3B), with range of 9 (NIES-298B) to 1,365 (FD4) in each genome. For the other cyanobacteria, similar patterns were observed despite the fact that their genome sizes and numbers of core and unique genes are different; however, the rate of change as more genomes are included was different between species (Fig. S1).

We then assessed the essentiality of the core genes by comparing them to the essential genes in Synechococcus elongatus, a photosynthetic model organism which has a much smaller genome (2.7 Mb) (19). We found a high degree of overlap between them: 73.4% and 84.0% of the essential genes were core and soft-core genes (present in ≥95% of the genomes), respectively. Because *S. elongatus* rarely forms blooms, this suggests that additional genes besides the core genes are needed for CyanoHABs. Next, we examined how many genes were involved in pathways for CyanoHABs, as curated in the pathway database CyanoPATH (12). A total of 185 core genes were found to be involved in CyanoPATH (Fig. 3C). Functionally, these genes are mainly involved in nitrogen utilization (24 genes), PS-I (photosystem-1) (20), CCM (CO_2_-concentrating mechanism) (21), AAPEP (amino acid utilization) (22), MAA (mycosporine-like amino acid biosynthesis) (22), and gas vesicle biosynthesis (14), followed by PBS (phycobilisome) (10) and pathways for utilizing other nutrients (23).

### The genes for CyanoHABs are under strong purifying selection.

**FIG 4:**
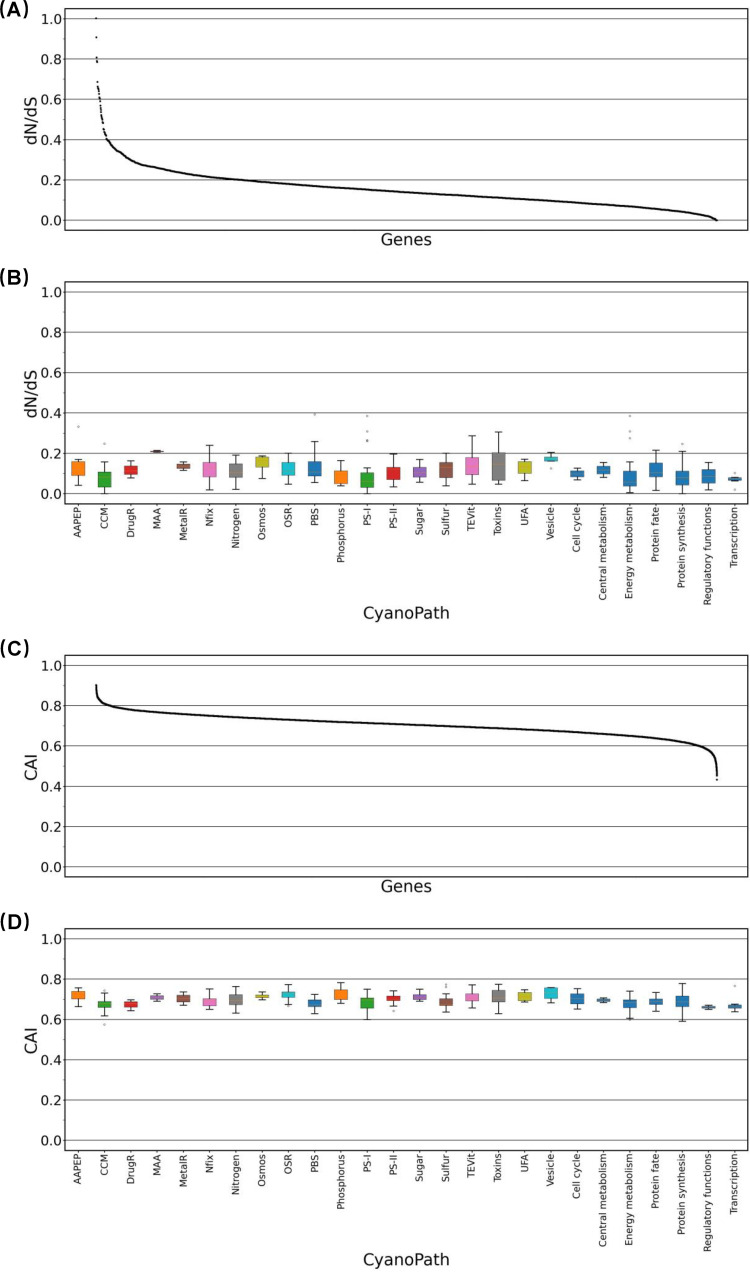
d*N*/d*S* and CAI for all homologous genes in 50 genomes of M. aeruginosa. (A) The d*N*/d*S* distribution of all genes. (B) The d*N*/d*S* of the genes in the pathways of CyanoPATH. (C) Distribution of CAI in all *M. aeruginosa* genes. (D) CAI for genes in different groups of functions. Purple bars are pathways strongly associated with CyanoHABs; green bars are pathways not specifically related to CyanoHABs; last 7 blue bars represent essential biological functions (19).

To determine whether bloom-forming cyanobacteria are under positive selection, we first performed a nonsynonymous/synonymous rate ratio (d*N*/d*S*) analysis. All homologous genes of the strains of each species had a d*N*/d*S* ratio of <1, with a median of about 0.32 and a minimum close to 0 (Fig. 4A and S2); this strongly suggests that the strains from different locations are under purifying selection, not positive selection for divergence (9, 21, 24). In *M. aeruginosa*, all d*N*/d*S* ratios are less than 0.85, with a median of 0.3. When the homologous genes were categorized into the functional pathways as curated in CyanoPATH (12), these functional genes showed similar d*N*/d*S* ratios to those of the housekeeping genes, e.g., central metabolism, transcription, translation, etc. (Kruskal-Wallis test, *P* > 0.5; Fig. 4B) (19). This strong purifying selection on the genes for CyanoHABs and housekeeping genes suggests that these genes are functionally important to the fitness of the species and any deleterious mutations are eliminated from the population.

Second, we determined the codon usage bias of the CyanoPATH genes in *M. aeruginosa*, a result of the balance between mutation and selection on translation. We used CAI (codon adaptation index), an index of adaptation in terms of the nonrandom synonymous codon usage of a gene from synonymous codons for translational efficiency (25). The CAI values ranged from 0.42 to 0.90, with a median of 0.75 (Fig. 4C). When the homologous genes are grouped into the metabolic pathways in CyanoPATH, their CAI values are similar to those in core metabolism and housekeeping processes (e.g., energy metabolism and protein) (Kruskal-Wallis test, *P* > 0.5; Fig. 4D).

### Correlation between gene expression and CAI and d*N*/d*S*.

**FIG 5:**
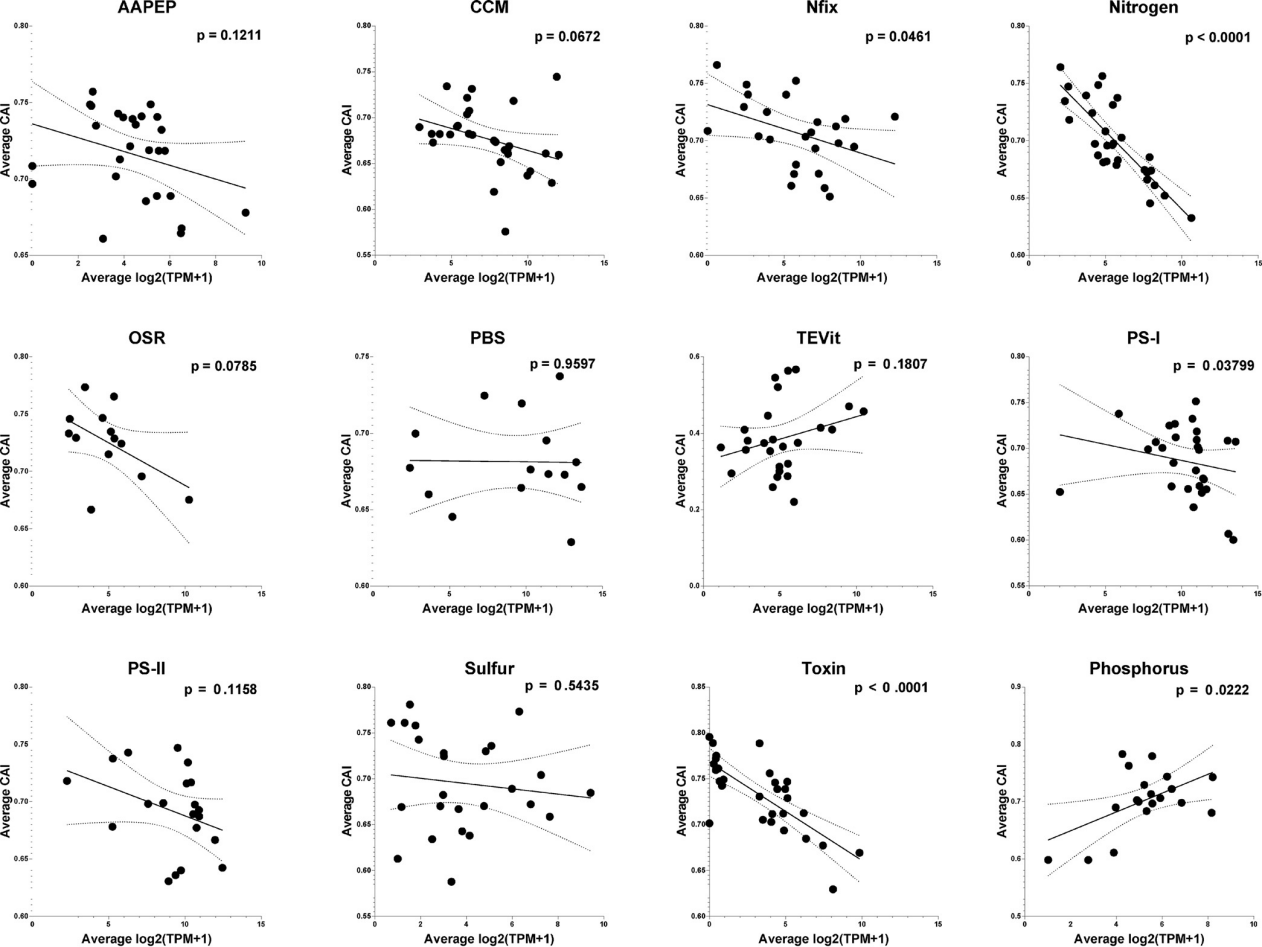
Relationships between gene expression and CAI in M. aeruginosa. A linear regression was drawn for each metabolic pathway with 95% confidence intervals and *P* values.

We further tested the possibility that the codon usage bias of genes can predict their expression level. Interestingly, the CAI in the common bloom-forming species *M. aeruginosa* was negatively correlated with the gene expression levels in most pathways in CyanoPATH, except for PBS, TEVit (vitamins and trace elements), and phosphorus utilization, using the metatranscriptomes from our study and 2 other studies (Fig. 5 and Table 1). Such negative correlations were similar in the 3 data sets used, with correlation coefficients ranging from 0.09 to 0.42 (*P* < 0.001). This suggests that the genes which were highly expressed in most of the pathways tended to have low CAI, while those with high CAI tended to be expressed at relatively low levels. Next, we analyzed the relationship between d*N*/d*S* and gene expression. A similar negative correlation was observed, but it was significant in fewer pathways (Fig. S3). Among these, only Nfix (nitrogen fixation), nitrogen utilization, TEVit, PS-I, cyanotoxin biosynthesis, and phosphorus utilization showed significant correlations (*P* < 0.05).

**TABLE 1:** Correlation coefficients between TPM and CAI in each metatranscriptome data set^*a*^

Data set	Correlation coefficient	No. of genes
Lake Erie 2012_1	−0.42	1,574
Lake Erie 2012_2	−0.09	1,577
Lake Erie 2012_3	−0.36	1,524
Lake Taihu 2019_1	−0.31	4,144
Lake Taihu 2019_2	−0.34	4,088
Lake Taihu 2019_3	−0.33	3,897
Kranji Reservoir 2014	−0.07	2,291

a TPM, transcripts per kilobase million; CAI, codon adaptation index.

### Relationship between SNP and gene expression.

**FIG 6:**
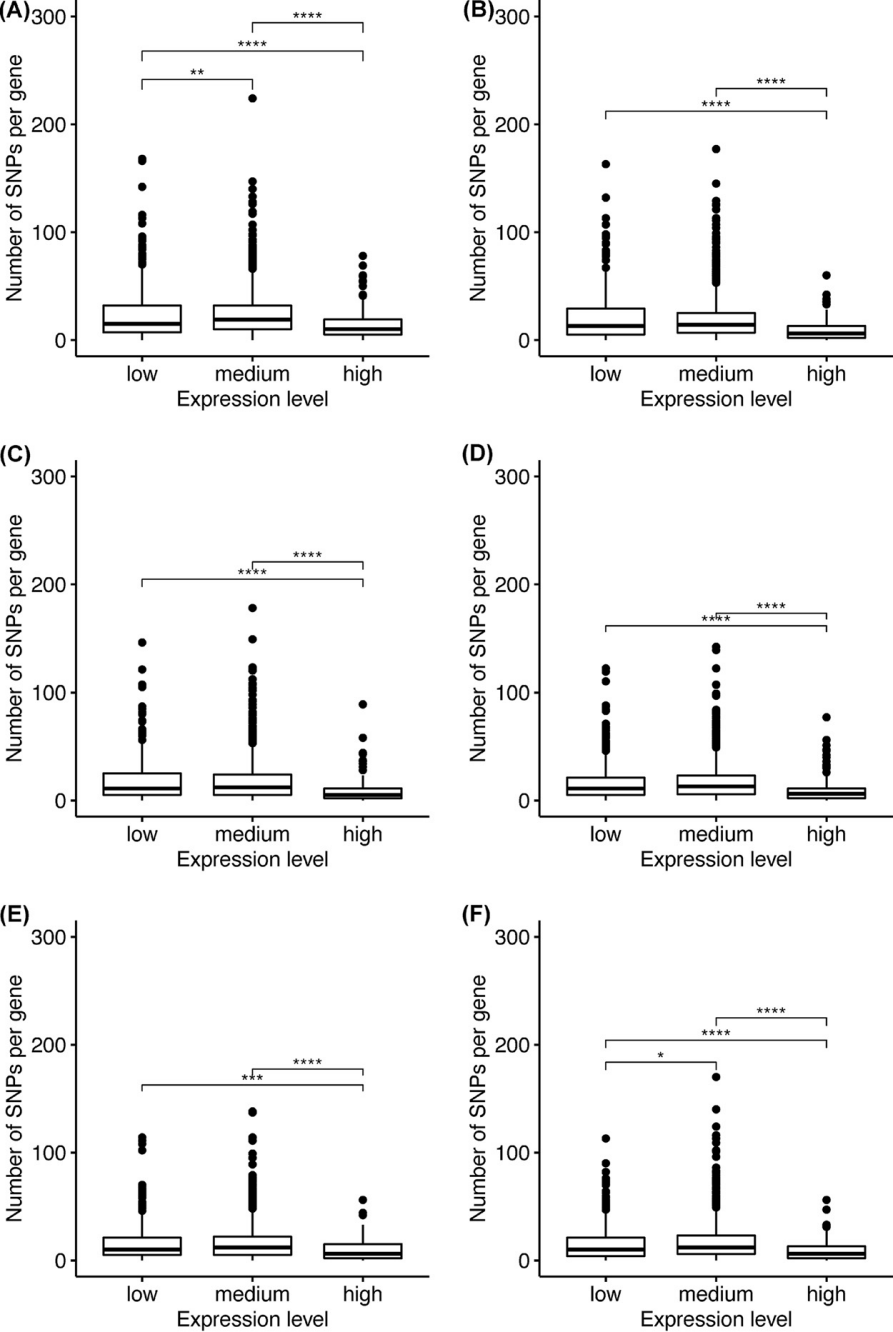
SNP numbers in 3 gene expression levels of *in situ* metatranscriptome data from Lake Taihu. Data Set 1: (A) SRA: SRR21035067, (B) SRA: SRR21035066. Data Set 2: (C) SRA: SRR21035065. (D) SRA: SRR21035064; Data Set 3: (E) SRA: SRR21035063, (F) SRA: SRR21035062. Kruskal-Wallis test: *, *P* < 0.05; ***, *P* < 0.01; ****, *P* < 0.001. Using the EMBL-EBI’s Expression Atlas thresholds as our guide, we set the following quantitative cutoffs for gene expression: low expression, 0.5 to 10 transcripts per kilobase million (TPM); medium, 11 to 1,000 TPM; high, >1,000 TPM (20).

Finally, we determined the single nucleotide polymorphisms (SNPs) in the genes of *M. aeruginosa*. A standard Poisson distribution of the SNPs was observed for all genes, using the Lake Taihu data sets (Fig. 6). About 2,500 genes had 5 to 50 SNPs, about 500 had more than 50 SNPs, and the rest (800) had very few SNPs. Moreover, all 1,580 core genes had SNPs, and 3,992 pan genes had at least 1 SNP. No linear correlations were observed between the number of SNPs and gene expression level. However, by dividing the gene expression levels into 3 groups, low (transcripts per kilobase million [TPM] = 0.5 to 10), medium (TPM = 11 to 1,000), and high (TPM > 1,000) (20), we found significant differences in the number of SNPs between the low and high groups and between the medium and high groups (Kruskal-Wallis test, *P* < 0.001). In other words, genes expressed at a high level had fewer SNPs than those expressed at a low or medium level (Fig. 6).

## DISCUSSION

CyanoHABs of dozens of species are prevalent in eutrophic waters worldwide. One key question as to this ubiquitous dominance is whether CyanoHABs result from positive selection by eutrophic waters in different geographic locations. Our results show that no directed (positive) evolution was observed and, instead, most genes are under strong purifying selection. Meanwhile, the phylogenies of conspecific strains from different geographic origins showed no clear divergent evolution patterns; instead, some of the geographically distant strains tended to cluster together. This discrepancy between phylogeny and geography has been repeatedly observed in other studies (26, 27). This common closeness between geographically distant strains shared in all 9 select cyanobacteria contradicts their geographic separation, which falsifies positive selection toward water eutrophication. Furthermore, the purifying selection on most genes provides the most important evidence against positive selection. Additional support for purifying selection comes from the similar d*N*/d*S* ratios between the pathways for CyanoHABs and central metabolism; these bloom-driving functions are no different in the selection regime than the essential housekeeping functions. Meanwhile, we are also aware that conventional d*N*/d*S* ratios used at the population level are developed for distinct sequences and thus caution should be taken when using them (28). Despite these considerations, these two findings provide evidence for purifying selection, not positive selection, in cyanobacteria.

The lack of evidence for positive evolution in this study underscores the fact that water eutrophication due to human activities is a recent shift in the water nutrient state (10). First, this upward shift is unforeseeable to cyanobacteria, so they cannot prepare for it ahead of time in terms of biological functions. Second, after eutrophication occurs, they slowly begin to adapt to elevated nutrients; however, the past 100 years do not appear to have been enough time to accumulate beneficial mutations, given that their mutation rates are similar to those in bacteria (29, 30), that the effective population size is reduced in asexual organisms (31) and that beneficial mutations are lost to annual bottlenecking (32) when they overwinter (33, 34). This is not to say that no adaptation whatsoever has occurred since water eutrophication, but the molecular signature of adaptive evolution is probably insignificant and evades our detection at the moment.

The finding that CyanoHABs are not a consequence of positive selection by water eutrophication provides a foundation for understanding the molecular genetics of bloom-forming cyanobacteria. One important discovery is the negative correlation between CAI and gene expression, with genes of low CAI (<0.5) having higher expression. CAI has a range of 0 to 1, with the lower bound (CAI = 0) when only the least frequent codons are used and the upper bound (CAI = 1) when the most frequent codons are used in a gene (35). It has been shown that a value greater than 0.5 indicates a high bias (36), a result of selection. Thus, the negative correlation indeed reflects the level of purifying selection, the potential to increase transcription/translation efficiency (37, 38). Specifically, most genes have CAI values of >0.65, which is higher than the neutral 0.5 and suggests that the codons are already used in a biased manner. Thus, the genes used the most, which are highly expressed, prefer common codons to facilitate transcription/translation and thus have high CAI, while the genes less used are not as highly expressed and can use rare codons to achieve an overall transcription efficiency. The negative relationship between the number of SNPs and gene expression levels can be understood in the same manner. Fewer SNPs reflect stronger purifying selection and the importance of certain genes which are often used and indeed expressed at higher levels than those with more SNPs.

The finding that CyanoHABs are not the results of positive selection by water eutrophication also has an important bearing on bloom control. A lack of positive selection suggests that bloom-forming cyanobacteria are functionally ready prior to water eutrophication. In other words, these species must have these functions encoded in their genomes which are also fully functional prior to water eutrophication so that when freshwaters became eutrophic (i.e., loaded with excess external nutrients from human activities), they can make the most of elevated nutrients more than co-living algae to dominate the phytoplankton community. In light of the superior biological functions in place in bloom-forming cyanobacteria, nutrient reduction appears to be the most effective method for controlling CyanoHABs because it eradicates the problem at the source; however, it is also the most expensive and time-consuming (39).

In summary, we found that CyanoHABs are not a result of positive selection by water eutrophication. Instead, bloom-forming cyanobacteria are under purifying selection. Consequently, the genes with relatively less codon usage bias and lower SNPs are expressed at higher levels than those with more codon bias and SNPs. Given the superior functions encoded by the genomes of bloom-forming cyanobacteria and the driving role of nutrients established by scientific consensus (10), this study also suggests that the most effective control of CyanoHABs is nutrient reduction in eutrophic waters.

## MATERIALS AND METHODS

### Genomes and sources.

In December 2020, all the genome sequences of 9 bloom-forming cyanobacteria (M. aeruginosa, Aphanizomenon flos-aquae, Arthrospira platensis, Cylindrospermopsis raciborskii, Fischerella thermalis, Nodularia spumigena, Planktothrix agardhii, Synechococcus elongatus, and Synechocystis salina) according to CyanoPATH were obtained from the National Center for Biotechnology Information (NCBI) genome database. Metagenome-assembled genomes (MAG) were filtered out because MAGs are mostly incomplete and may contain fragments from other species due to practical or technical limitations (40). Only the genomes reconstructed from pure cultures (single isolates) were subject to the following analyses, and their features are listed in Data Set S1 with the geographic locations of samples found listed by the NCBI, Joint Genome Institute (JGI), National Institute for Environmental Studies (NIES), and Biological Resource Center of Institut Pasteur (CRBIP).

### Comparative genome analyses (core-pan-genome analysis).

All the genomes were annotated with Prokka v1.14.6 (41) using the corresponding genus databases to determine the location and attributes of the genes they contained. The core-pan-genome analysis was performed with Roary v3.13.0 using the default settings (42). The resulting core- and pan-genomes were visualized by a core-pan-genome plot using Prism v9.1.1 (43) and a flower plot using R v4.1.1 (44).

### Phylogenetic analyses.

A whole genome-based phylogenetic tree of all cyanobacteria was built using PhyloPhlAn v3.0.60 in “medium diversity” mode (45) and was visualized on iTOL (46).

For M. aeruginosa, 2 phylogenetic trees were reconstructed based on the core genome and whole genome sequences, respectively. The core genome sequences of 50 M. aeruginosa strains generated from pangenomic analysis were aligned with Mafft v7.310 using the FFT-NS-2 progressive algorithm (47). A Maximum Likelihood (ML) tree was built by FastTree v2.1.10 with default parameters (48) and visualized online on iTOL (46). The genome sequences of M. aeruginosa were then searched against microcystin synthetase A (GenBank accession no. AAF00960.1) with NCBI BLAST+ v2.6.0 (49), and those containing the enzyme were considered toxic (50).

The whole genome sequences of 50 M. aeruginosa, P. agardhii (GenBank assembly no. GCA_003609755.1) and *Anabaena cylindrica* (GenBank assembly no. GCA_000317695.1) were uploaded to TYGS for genome-based phylogenetic analysis (22). The resulting phylogenetic tree was then uploaded to iTOL for visualization (46). The global distribution of the samples was visualized on a world map obtained from OpenStreetMap (51), and strains sharing the earliest common ancestor were lined using Tableau v2021.3 (18).

### Selection pressure analyses.

The d*N*/d*S* and CAI were calculated to determine the selection pressure on the protein-coding genes. Genes shared by less than 4 strains in each species and those without nonsynonymous mutations were excluded from the selection pressure analyses (52). The d*N*/d*S* of each gene was calculated using CodeML from the PAML (Phylogenetic Analysis by Maximum Likelihood) package v4.9j with default settings (53). For M. aeruginosa, random subsampling of 10 genomes was repeated 40 times. Then, d*N*/d*S* were calculated for each repetition and the average values were recorded.

The CAI values were determined by CAIcal v1.4 (25) based on the codon usage table available in the Codon Usage Database (54).

### Metatranscriptomic sequencing and analyses.

Water samples were collected from 3 locations at Lake Taihu with 2 biological replicates and processed for next-generation sequencing (NGS) as previously described (55). The prepared NGS libraries were loaded to the Illumina Mi-Seq platform for 150-bp paired-end sequencing. Two or three sets of RNA-seq data were first merged according to their sampling sites as shown in Table 1. Low-quality reads were filtered out using Trimmomatic v0.39 (56). The trim settings used are as follows: headcrop = 10, trailing = 20, slidingwindow = 4:26, minlen = 75. From the 50 M. aeruginosa strains, GenBank assembly no. GCA_002282945.1 was selected as the reference for the following analyses and annotated by Prokka v1.13 (41). Next, for each set of RNA-seq data, expression levels were calculated using RSEM v1.3.3 (23). Transcript abundances were quantified in terms of TPM.

The correlation coefficients between TPM and CAI values in each data set (Lake Erie, Lake Taihu and Kranji Reservoir) were calculated using the “corrr” package v0.4.3 (57).

### Genetic variant analyses.

Three sets of RNA sequencing data were used for SNP calling. We performed a two-pass alignment to map reads to the reference assembly (GenBank assembly no. GCA_002282945.1) using STAR v2.7.10a (58). Identical reads were marked using the MarkDuplicates tool in Picard v2.21.9 (59) and were ignored in downstream analysis. VarScan v2.4.4 was used to call variants with a minimum coverage value of 1 and a *P* value of 0.95 (60).

### Metabolic analyses.

A previous study identified 718 essential genes necessary for survival in cyanobacteria (19). Genes in our study which overlapped with the essential gene set were annotated as putative essential genes. Core genes were grouped into different metabolic pathways by searching them against a reference set of protein sequences obtained from CyanoPATH (11), a database of pathways associated with cyanobacterial blooms, using DIAMOND v0.9.14 (61). Those with less than 75% identity or less than 75% coverage (hit length divided by the reference length) were filtered out. Box plots of d*N*/d*S* and CAI values of core genes in each pathway were generated using the Python Matplotlib package v3.4.3 (62). Scatterplots of CAI versus transformed TPM values of core genes in each pathway were drawn by Prism v9.11 (43). TPM values were transformed into log(TPM + 1).

### Data and code availability.

The raw metatranscriptomic sequencing data generated from this study have been deposited in the NCBI Sequence Read Archive (SRA) under BioProject accession no. PRJNA869295. The SRA and GenBank accession numbers of the omics data used are listed in Table 2 and Data Set S1, respectively. All codes are available on request.

**TABLE 2:** Metatranscriptome data sets used in this study

Data set	Source	SRA^*a*^ accession no.
Lake Erie 2012_1	Steffen et al. (63)	SRR1927216, SRR1927217, SRR1927219
Lake Erie 2012_2	Steffen et al. (63)	SRR1927220, SRR1927222, SRR1927223
Lake Erie 2012_3	Steffen et al. (63)	SRR1927225, SRR1927229, SRR1927239
Lake Taihu 2019_1	This paper	SRR21035067, SRR21035066
Lake Taihu 2019_2	This paper	SRR21035065, SRR21035064
Lake Taihu 2019_3	This paper	SRR21035063, SRR21035062
Kranji Reservoir 2014	Penn et al. (55)	SRR1171050, SRR1171059, SRR1171061

a SRA, Sequence Read Archive (https://www.ncbi.nlm.nih.gov/sra).
