# Supplementary material for: Cyanobacterial Blooms Are Not a Result of Positive Selection by Freshwater Eutrophication
Source: Microbiol Spectr. 2022 Nov 29;10(6):e03194-22. doi: 10.1128/spectrum.03194-22 (PMC9769789; doi:10.1128/spectrum.03194-22)
Supplement: Supplemental file 1 — Fig. S1 to S3. Download spectrum.03194-22-s0002.pdf, PDF file, 0.9 MB [file spectrum.03194-22-s0002.pdf]

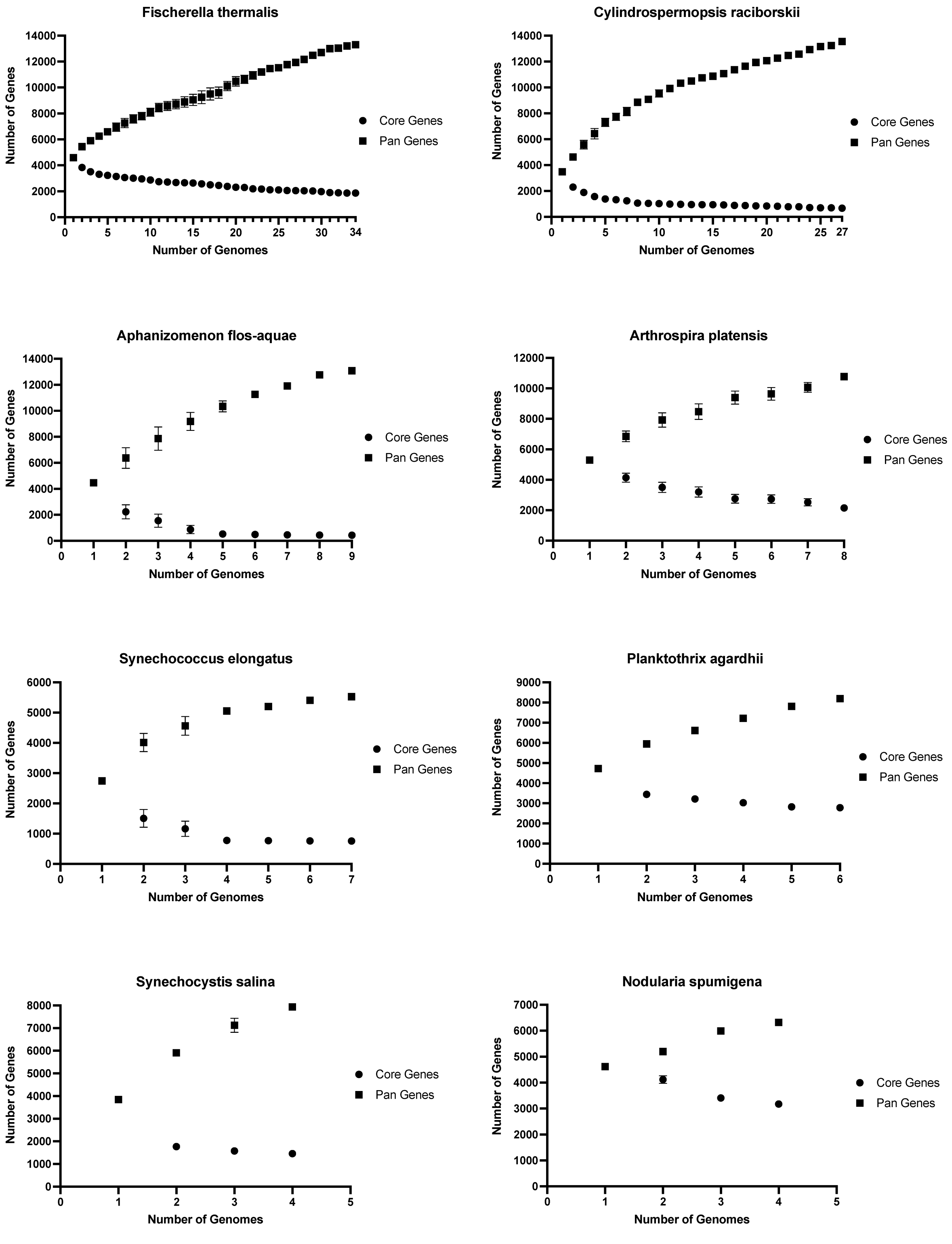

FIG S1. Core-pan-genome profiles of 8 bloom-forming cyanobacteria genomes. The colored boxes denote the pan-genome (square) and core genome (circle) sizes respectively with an increasing number of genomes.

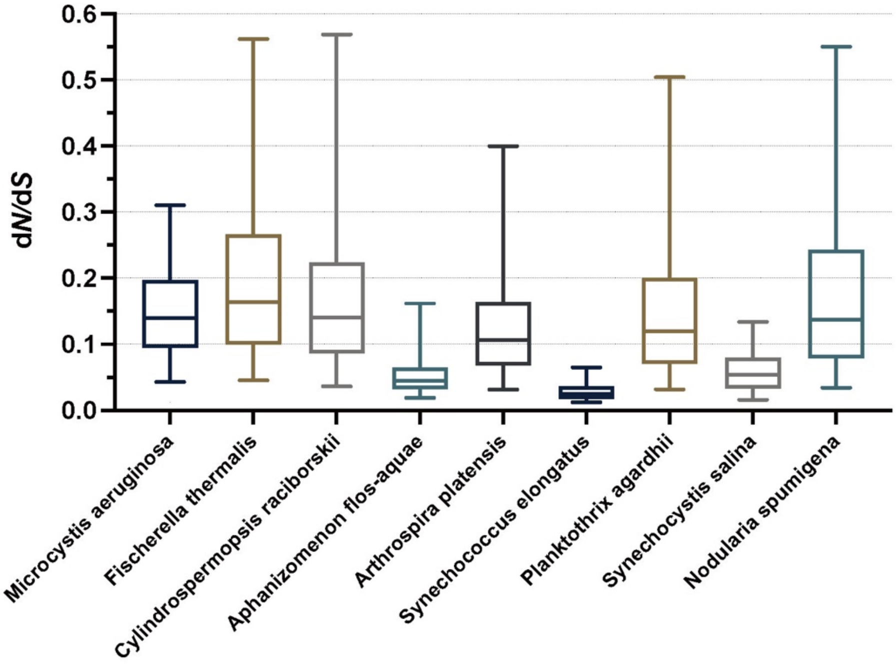

FIG S2. Box plot showing d*N*/d*S* for all genes in 9 bloom-forming cyanobacteria. Within each box, horizontal lines denote median values. Upper and lower limit of boxplot represent 75 th and 25th percentile. Whiskers represent 5th to 95 th percentile.

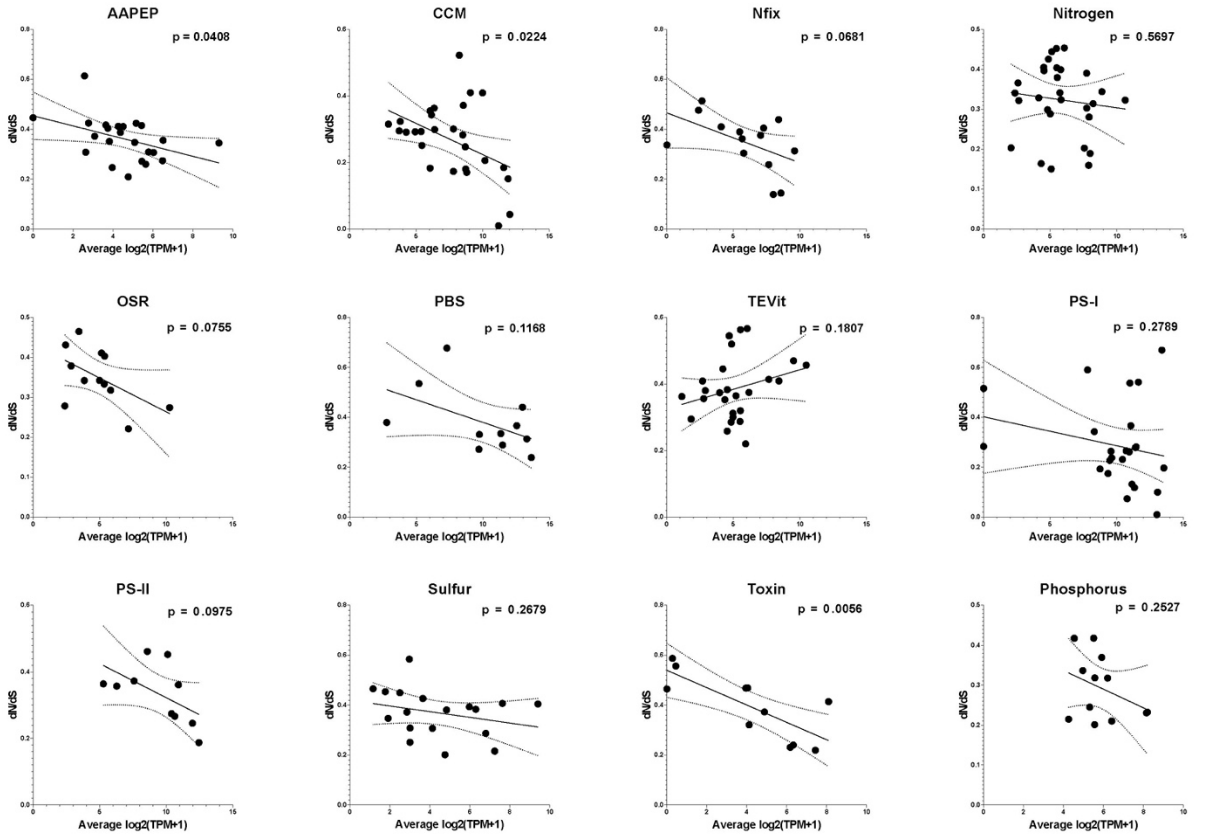

FIG S3. The relationships between gene expression and d*N*/d*S* in *Microcystis aeruginosa*. The linear regression was drawn for each metabolic pathway with 95% confidence intervals and P values were reported. AAPEP: uptake of amino acids and peptides; CCM: CO_2 concentrating mechanism; Nfix: Nitrogen fixation; Nitrogen: Nitrogen utilization; OSR: Redox restress resistance; PBS: Phycobilisome; TEVit: Trace element and vitamin uptake; PS-I: Photosystem I; PS-II: Photosystem II; Sulfur: Sulfur utilization; Toxin: Cyanotoxin biosynthesis; Phosphorus: Phosphorus utilization.
